# Dysfunction of low-density neutrophils in peripheral circulation in patients with sepsis

**DOI:** 10.1038/s41598-021-04682-x

**Published:** 2022-01-13

**Authors:** Ran Sun, Jiamin Huang, Yunxi Yang, Lu Liu, Yiming Shao, Linbin Li, Bingwei Sun

**Affiliations:** 1 Department of Burns and Plastic Surgery, Affiliated Suzhou Hospital of Nanjing Medical University, Suzhou, 215002 Jiangsu Province China; 2 School of Medicine, Jiangsu University, Zhenjiang, 212001 Jiangsu Province China

**Keywords:** Immunology, Diseases

## Abstract

Low-density neutrophils (LDNs) have been described in tumors and various autoimmune diseases, where they exhibit immune dysfunction and alter disease progression. Nevertheless, LDNs have been rarely reported in sepsis. We studied sepsis patients admitted to the intensive care unit. Wright-Giemsa stain assay and Transmission electron microscopy were performed to detect the morphology of neutrophils. Flow cytometry was used to analyze the number and function of LDNs. Concentration of cytokines was measured using ELISA. Neutrophil chemotaxis was examined using an under-agarose chemotaxis model. We found that LDNs were significantly elevated in patients with sepsis. Phenotypes and morphological characteristics suggest that LDNs may be formed by mixtures of neutrophils at various maturation stages. In vitro experiments showed that LDN formation was closely associated with neutrophil degranulation. We preliminarily discussed changes in immune function in LDNs. Compared with high-density neutrophils, expression levels of CXC chemokine receptor 4 on LDN surfaces were increased, phagocytotic capacity was decreased, and life span was prolonged. The chemotactic ability of LDNs was significantly reduced, possibly related to the increased expression of P2X1. These data suggest that LDNs are essential components of neutrophils in sepsis. To clarify the source and dysfunction mechanism of LDN in sepsis may be helpful for the diagnosis and treatment of sepsis in the future.

## Introduction

Sepsis is a severe complication of burns, trauma, and infections. It is also a significant cause of shock and multiple organ dysfunction syndrome^1^. Despite intense research into sepsis, morbidity and mortality have not improved significantly. The new definition of sepsis suggests that dysregulation of the host's immune response to infection should be recognized promptly because the loss of organ dysfunction can significantly diminish survival^2^. Several lines of evidence suggest that neutrophils participate in the immune response during sepsis^3,4^.

Neutrophils are still largely defined by their morphology. By contrast, immune cells such as lymphocytes and macrophages are divided into several subsets that exhibit different functional characteristics^5,6^. The heterogeneity of neutrophils has been reported more in recent years. In the physiological state, the heterogeneity of neutrophils derives from senescence and regeneration. Neutrophils have a short life span and rapidly age in peripheral circulation and tissues. These senescent neutrophils exhibit high levels of expression of CXC chemokine receptor 4 (CXCR4), CD11b, and CD49 and low levels of expression of CD62L. Phagocytosis and neutrophil extracellular traps (NETs) production in senescent neutrophils is enhanced^7^. These cells promote intravascular inflammation and regulate the stem cell microenvironment^7,8^. Unlike with the physiological state, the heterogeneity of neutrophils under pathological states has been studied more extensively. Tumor-associated neutrophils are characterized by varying subtypes under the influence of tumor microenvironment, producing "N1" and "N2" neutrophils. The former inhibits tumor growth, and the latter promotes tumor growth and metastasis^9^.

Low-density neutrophils (LDNs) were first identified in peripheral blood mononuclear cells (PBMCs) of patients with systemic lupus erythematosus (SLE) using the Ficoll-Hypaque density gradient centrifuge technique (Hacbarth et al.)^10^. In more studies on cell function, LDNs is a pro-inflammatory neutrophil that is characterized by increased IFN-γ and TNF-α expression, increased endothelial cytotoxicity and reduced phagocytosis capacity^11,12^. Unlike normal neutrophils, LDNs contain cells with myelocyte-like or band-shaped nuclei characteristic of immature granulocytes in addition to cells with multilobed nuclei characteristic of mature neutrophils^13,14^. Studies on cancer^14,15^ showed that there are at least three subsets of neutrophils in circulation: immature LDNs, mature LDNs, and mature high-density neutrophils (HDNs). The dynamic transformation between subsets may be responsible for the increase in the number of neutrophils in PBMCs. LDNs were found in pregnant women, and this subset of neutrophils overexpressed arginase 1. The depletion of arginine resulted in blocked T cell signaling and suppressed function, protecting the fetus from immune rejection^16^. Other studies showed that LDNs occur in a variety of diseases where they positively correlated with disease severity^17–19^. Nevertheless, it remains unclear whether LDNs lead to the disease's progression or represent an epiphenomenon.

Although LDNs have been extensively studied in various diseases, the presence of LDNs in sepsis does not appear to have received significant attention. Therefore, in the current study, we determined the frequency and characteristics of LDNs in patients with sepsis and conducted a preliminary investigation into the origin of LDNs in sepsis using a lipopolysaccharide (LPS)-stimulated system. We also investigated abnormalities of LDNs immune function and phenotypic differences in sepsis.

## Materials and methods

### Ethical approval

The Ethics Committees of the Suzhou Hospital Affiliated to Nanjing Medical University approved the study. Blood specimens were extracted from the cubital veins of healthy volunteers and sepsis patients. Written informed consent was received from participants before the study began. We confirmed that all experiments were performed in accordance with the relevant guidelines and regulations.

### Human subjects

We studied prospectively enrolled sepsis patients admitted to the intensive care unit of Affiliated Suzhou Hospital of Nanjing Medical University between February 2020 and December 2020. Patients aged > 18 years, diagnosed with sepsis, and had sequential organ failure assessment (SOFA) scores ≥ 2 were eligible for the study. We excluded patients who were pregnant, had hematologic diseases, or hematopoietic dysfunction that affected neutrophils or used leucocyte-raising drugs in the previous week. We recruited 15 healthy control volunteers. During the follow-up period, clinical and biological data were collected. The data collection included demographic characteristics (age, gender); complete blood count (WBC count, neutrophil; lymphocyte; neutrophil–lymphocyte count ratio, NLCR); inflammatory biomarkers (procalcitonin, PCT, C-reactive protein, CRP); site of infection; and microbiological findings. We recorded the initial severity assessed by the APACHE II score (range 0–71) and the SOFA score (range 0–24) at admission.

### Isolation of human neutrophils

Venous blood was obtained from healthy volunteers and sepsis patients. Neutrophils were isolated as previously described^20^. Briefly, erythrocytes were precipitated with 3% dextran, and mononuclear cells were removed using Ficoll-Paque density centrifugation. A high-density fraction pellet containing HDNs was further subjected to red blood cell lysis. The low-density fraction (PBMC) containing the LDN was collected into phosphate-buffered saline + 0.5% bovine serum albumin. LDNs were further purified using an EasySep human neutrophils enrichment kit (StemCell Technologies). Neutrophils were suspended in HBSS at 1.0 × 10^7^ cells/ml. The survival rate and purity of neutrophils were greater than 95%.

### Wright–Giemsa stain assay

Neutrophilic granulophilic smears were prepared. After natural drying, Wright-Giemsa solution (SolarBio) (2–3 drops) was added to cover the entire specimen smear and incubated at room temperature for 1–2 min. We added phosphate buffer (pH 6.4) and mixed it by shaking slides with Wright-Giemsa solution thoroughly. Dying lasted 3–5 min. The Giemsa solution was discarded, followed by washing in water. The results of cell staining were observed using an inverted microscope.

### Transmission electron microscopy

Neutrophils were detected using transmission electron microscopy as described previously^14,20^. Briefly, neutrophils were fixed in 2% glutaraldehyde followed by 1% osmium tetroxide. The cells were dehydrated in graded ethanol solutions and embedded in epon. The blocks were sectioned using an ultracut (Leica UC7), and sections of 80 nm were obtained and stained with uranyl acetate and lead citrate. Ultrathin sections were photographed using an HT-7700 electron microscopy (Hitachi).

### Flow cytometry

Neutrophils were suspended in ice-cold phosphate-buffered saline with 1% fetal bovine serum at 2 × 10^6^ cells/ml. Antibodies used in the experiment include CD66b (G10F5, BD Pharmingen, USA), CD10 (HI10α, Biolegend, USA) CXCR4 (12G5, Biolegend, USA) and P2X1 (APR-022-F, Alomone labs, Israel). Apoptosis was quantified by annexin V-PE and propidium iodide (PI) staining, with early apoptotic cells being annexin V^+^/PI^-^ and late apoptotic cells annexin V^+^/PI^+^. We counted the proportion of all apoptotic neutrophils (annexin V^+^). Antibodies or apoptosis kits were used according to the manufacturer’s instructions. After incubating for 20 min at 4 °C in the dark, cells were washed and measured using a FACS Canto II cytometer (BD Biosciences). The data were analyzed using FlowJo V10 (Ashland, OR, USA).

### In vitro neutrophil transition

HDNs were isolated from healthy volunteers and incubated for 3 h at 37 °C. This experiment was done in the presence or absence of LPS. Following incubation, HDNs and LDNs were isolated, and the distribution was evaluated using FACS. Cell culture supernatants were collected, the concentration of myeloperoxidase (MPO) and matrix metallopeptidase 9 (MMP-9) was quantified using enzyme-linked immunosorbent assays (MultiSciences, Hangzhou, China).

### Neutrophil phagocytosis

According to the manufacturer's instructions, phagocytosis capacities of neutrophils were measured using a pHrodo *E. coli* BioParticles Phagocytosis kit (Thermo Fisher Scientific, USA). Neutrophils were diluted to 1 × 10^6^ cells/mL buffer and incubated with the pHrodo *E. coli* particles at 37 °C and 5% CO_2_ for 0.5 h. Then, samples were centrifuged for 5 min. The phagocytosis of neutrophils was measured using flow cytometry.

### Measurement and analysis of neutrophils chemotaxis

An under-agarose neutrophil chemotaxis model was established as previously described^21^**.** Briefly, an agarose solution (1.2%) was boiled and mixed with a heated medium (HBSS and RPMI 1640 medium containing 20% heat-inactivated fetal bovine serum). Approximately 3 ml of the mixed solution was poured into a 35-mm culture dish and cooled gently. After the agarose solidified, three wells, 3.5 mm in diameter and 2.8 mm apart, were cut into a straight line in the gel. The middle well was filled with N-methionine leucine phenylalanine (fMLP, 0.1 µmol, 10 µl) and the side wells were filled with neutrophils (1.0 × 10^7^/mL, 10 µl). Gels were incubated for 2 h in a 37 °C/5% CO_2_ incubator. The original patterns of neutrophil chemotaxis were observed using a microscope (OLYMPUS IX71). We developed software for cell chemotaxis analysis that divided the chemotaxis area into three zones. We defined chemotactic distance (CD, μm), chemo cell ratio (CCR) and chemo index (CI), to evaluate the chemotactic function of neutrophils more comprehensively.

### Statistical analyses

GraphPad Prism 8.0 was used for statistical analysis. Continuous data were tested for normality using the Shapiro–Wilk test. All results were expressed as mean ± SD. One-way factorial analysis of variance (ANOVA) and the Student’s t-test for comparisons were performed. *P* < 0.05 was considered significant.

## Results

### Baseline characteristics of the study population

We recruited 26 patients with sepsis and 15 healthy controls. The baseline clinical and laboratory characteristics of the enrolled participants are displayed in Table 1. There were no significant differences between sepsis patients and healthy controls concerning age or gender.

**Table 1:** Characteristics of healthy controls and patients with sepsis.

Characteristics	Sepsis	Healthy controls	*P* value
N	n = 26	n = 15	
Age	69(37–85)	64(34–79)	0.16
Sex, male (%)	15(57.7)	7(46.7)	0.4953
WBC count(10^9^/L)	13.96 ± 1.27	6.41 ± 0.30	< 0.0001
Neutrophil(10^9^/L)	11.66 ± 1.24	3.52 ± 0.30	< 0.0001
Lymphocyte(10^9^/L)	1.21 ± 0.14	2.04 ± 0.12	0.0003
NLCR	15.18 ± 2.85	1.56 ± 0.15	0.0009
PCT(ng/ml)	5.25 ± 3.23	N/A	
BNP(pg/ml)	2311 ± 474.9	N/A	
CRP(mg/L)	112 ± 45.26	N/A	
APACHE II score	16(9–24)	N/A	
SOFA score	4(2–7)	N/A	
Site of infection, n (%)			
Pulmonary	14(53.8)	N/A	
Abdominal	4(15.4)	N/A	
Other	8(30.8)	N/A	
Microbiologically documented, n (%)			
Gram-positive bacteria	9(34.6)	N/A	
Gram-negative bacteria	12(46.2)	N/A	
None cultured	5(19.2)	N/A	

Data presented as number (percentage) or mean (standard deviation).

*WBC* white blood cell, *NA* not applicable, *NLCR* neutrophil–lymphocyte count ratio, *PCT* procalcitonin, *BNP* B type natriuretic peptide, *CRP* C-reactive protein.

### Distinct circulating neutrophil subpopulations in sepsis

**Figure 1:**
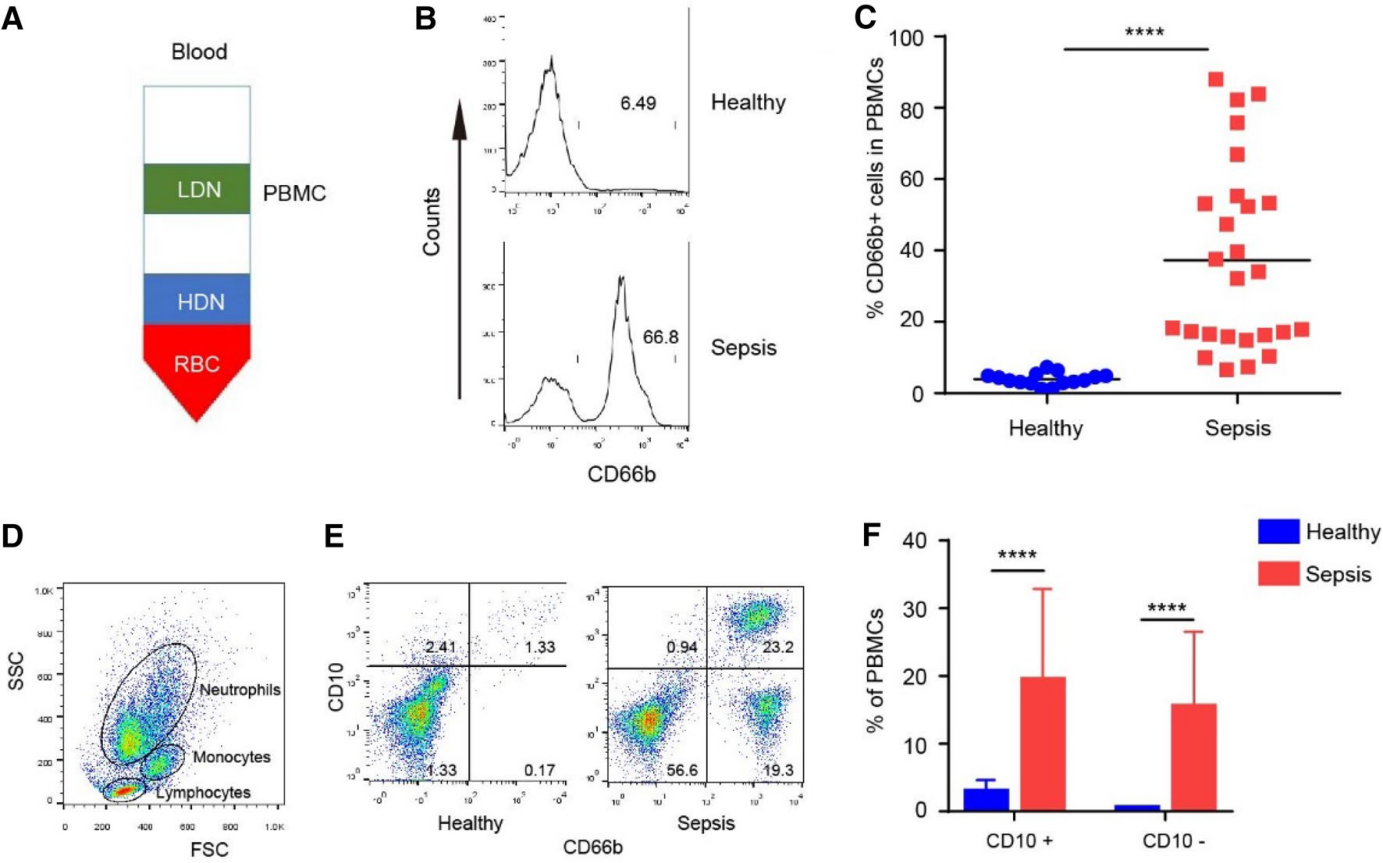
Heterogeneous LDNs in peripheral circulation in sepsis patients. (**A**) Complete venous blood was extracted, and density gradient centrifugation was performed to isolate PBMCs (containing LDNs), granulocytes (primarily HDNs), and erythrocytes. (**B**, **C**) The percentage of LDNs (CD66b^+^ cells) in healthy volunteers and sepsis patients was analyzed using flow cytometry. (**D**) Flow cytometric gating of various cells in the PBMCs. (**E**) CD66b and CD10 were labeled simultaneously and measured using flow cytometry. (**F**) Proportion of CD10^+^ LDNs and CD10^-^ LDNs in PBMCs.

In recent years, studies on neutrophils' heterogeneity have been increasing. We collected the peripheral blood of sepsis patients and purified neutrophils using density gradient centrifugation (Fig. 1A). CD66b is strongly expressed by neutrophils. In this study, we found that a high proportion of CD66b-neutrophils were found in PBMCs. In patients 3, 16, and 21, CD66b-positive neutrophils were the main component of PBMCs (87.93%, 82.20% and 83.90%, respectively). In healthy volunteers, the proportion of LDNs was very low (3.96 ± 0.4% vs. 37.3 ± 5.1%, Fig. 1B,C). Gating strategy using flow cytometry showed that PBMCs in sepsis patients included neutrophils, monocytes, and lymphocytes (Fig. 1D). There were substantial differences in the proportions of cells among patients.

Next, we tested the hypothesis that these cells formed various subsets by identifying LDN phenotypes. CD10 is a marker of mature neutrophils. Using simultaneous labeling of CD66b and CD10, we found that the majority of HDNs were CD66b^+^CD10^+^, while in PBMCs, CD66b^+^CD10^+^ occurred 19.29 ± 2.7% and CD66b^+^CD10^−^ occurred in 15.33 ± 2.2% (Fig. 1E,F). LDNs were composed of two subsets of neutrophils. These results suggest that LDNs composed of mature and immature neutrophils are produced during sepsis. The expression of CD10 in PMNs can down-regulate inflammation and is correlated with the severity of sepsis. Therefore, the low expression of CD10 in PMN of sepsis patients may indicate disease progression or changes in inflammation.

### Morphological characteristics of LDNs in sepsis

**Figure 2:**
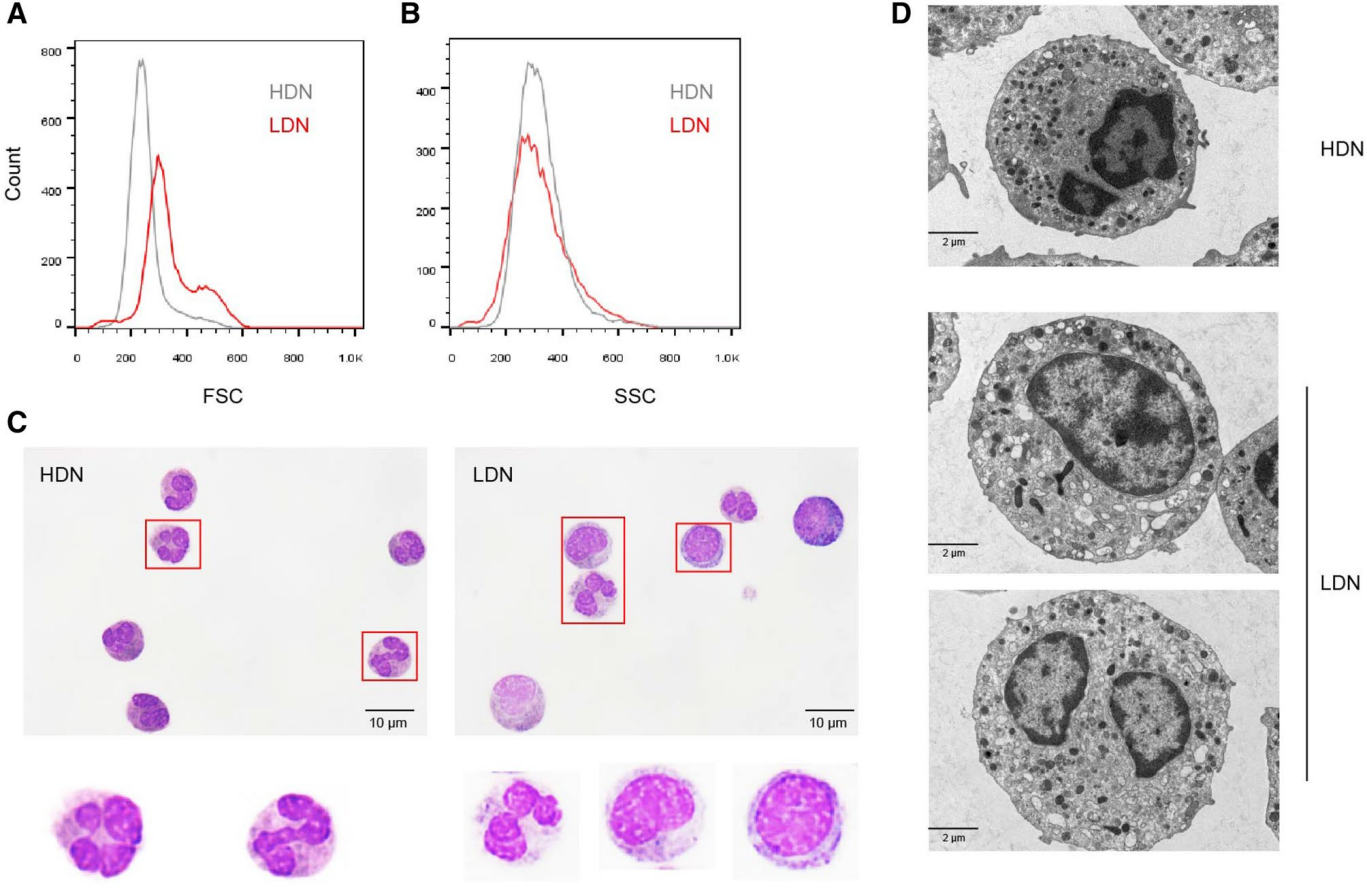
Morphological characteristics of LDNs in sepsis patients. (**A**, **B**) FACS analysis of LDNs (red) and HDNs (gray) isolated from a sepsis patient showing that LDNs have increased FSC with no change in SSC. (**C**) Representative Wright stain images of HDNs and LDNs. (**D**) Representative transmission electron micrographs of HDNs and LDNs.

Next, we performed morphological analysis on HDNs and LDNs. Flow cytometry analysis revealed that there were two cell subsets of different sizes in LDNs. LDNs had higher forward scatter (FSC) than HDNs with similar side scatter (SSC) (Fig. 2A,B). Wright–Giemsa stain revealed that HDNs appear to be a homozygous population of mature, multilobed nuclei neutrophils. LDNs are heterogeneous populations, consisting of myelocyte-like or band-shaped nuclei and immature neutrophils (Fig. 2C). Previous studies suggested that the increase in LDNs may be due to the activation of HDNs by various stimuli and subsequent degranulation^14,22^. To determine whether LDNs result from activated degranulation or an increased proportion of immature phenotypes, we compared their ultrastructural characteristics with those of HDNs using transmission electron microscopy. We found that, compared with granulocytes of normal density, LDNs showed less nuclear lobulation, some cells showed increased intracellular vacuoles and reduced granules, and some cells showed similar granule numbers as HDNs (Fig. 2D). This suggests that the source of LDNs may be partly related to neutrophil degranulation.

### Degranulation mediated the transition from HDN to LDN in sepsis

**Figure 3:**
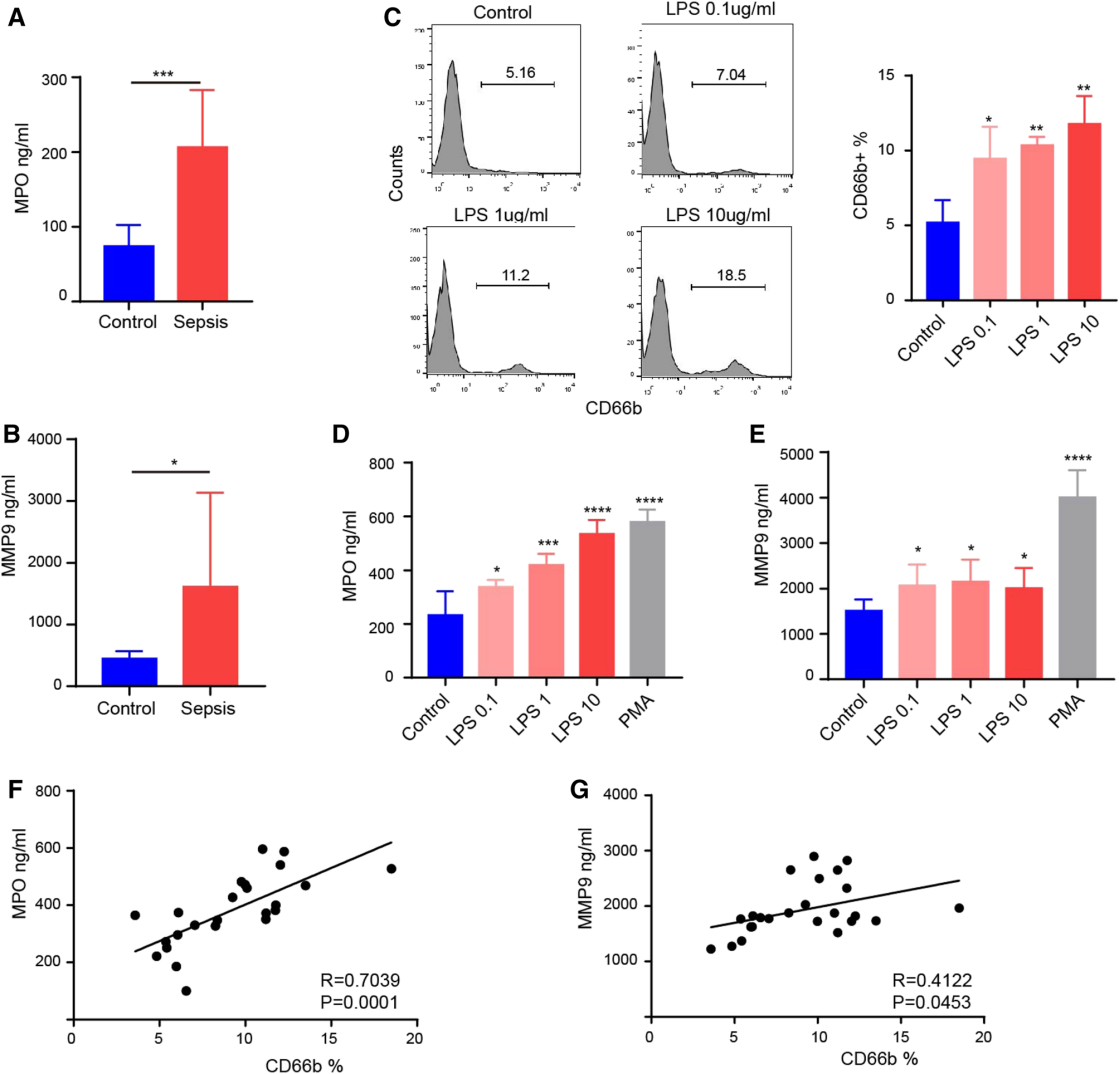
Degranulation is associated with the production of LDNs. (**A**) MPO concentrations in healthy controls and sepsis patients. (**B**) MMP9 concentrations in healthy controls and sepsis patients. (**C**) Changes of LDNs in PBMC layer after stimulation of neutrophils with various concentrations of LPS. (**D**) MPO concentration in the supernatants of neutrophils stimulated by LPS or PMA. (**E**) MMP9 concentration in the supernatants of neutrophils stimulated by LPS or PMA. (**F**, **G**) Correlation analysis of LDNs ratio and degranulation components.

To explore the source of LDNs in sepsis, we first measured degranulation indicators in the serum of sepsis patients. We found that levels of MPO (Fig. 3A) and MMP9 (Fig. 3B) were significantly higher than those of healthy volunteers. We simulated sepsis in vitro and tested the proportion of LDNs that could be affected by cultured whole blood cells (after erythrocyte sedimentation) in the presence of elevated LPS concentrations. We observed a dose-dependent increase in the proportion of CD66b-positive neutrophils in PBMC fraction after LPS stimulation (Fig. 3C), while the increase of MPO (Fig. 3D) and MMP9 (Fig. 3E) in the cell culture supernatants were roughly in line with the increasing trend of LDNs. We used phorbol myristate acetate (PMA) as a stimulant and found that it strongly induced neutrophil degranulation. We also observed a correlation between LDN ratios and MPO/MMP9 concentrations in an in vitro incubation system. The LDNs ratio positively correlated with MPO/MMP9 concentration, and the results of MPO were more obvious (Fig. 3F,G).

### LDN showed obvious abnormal immunological function

**Figure 4:**
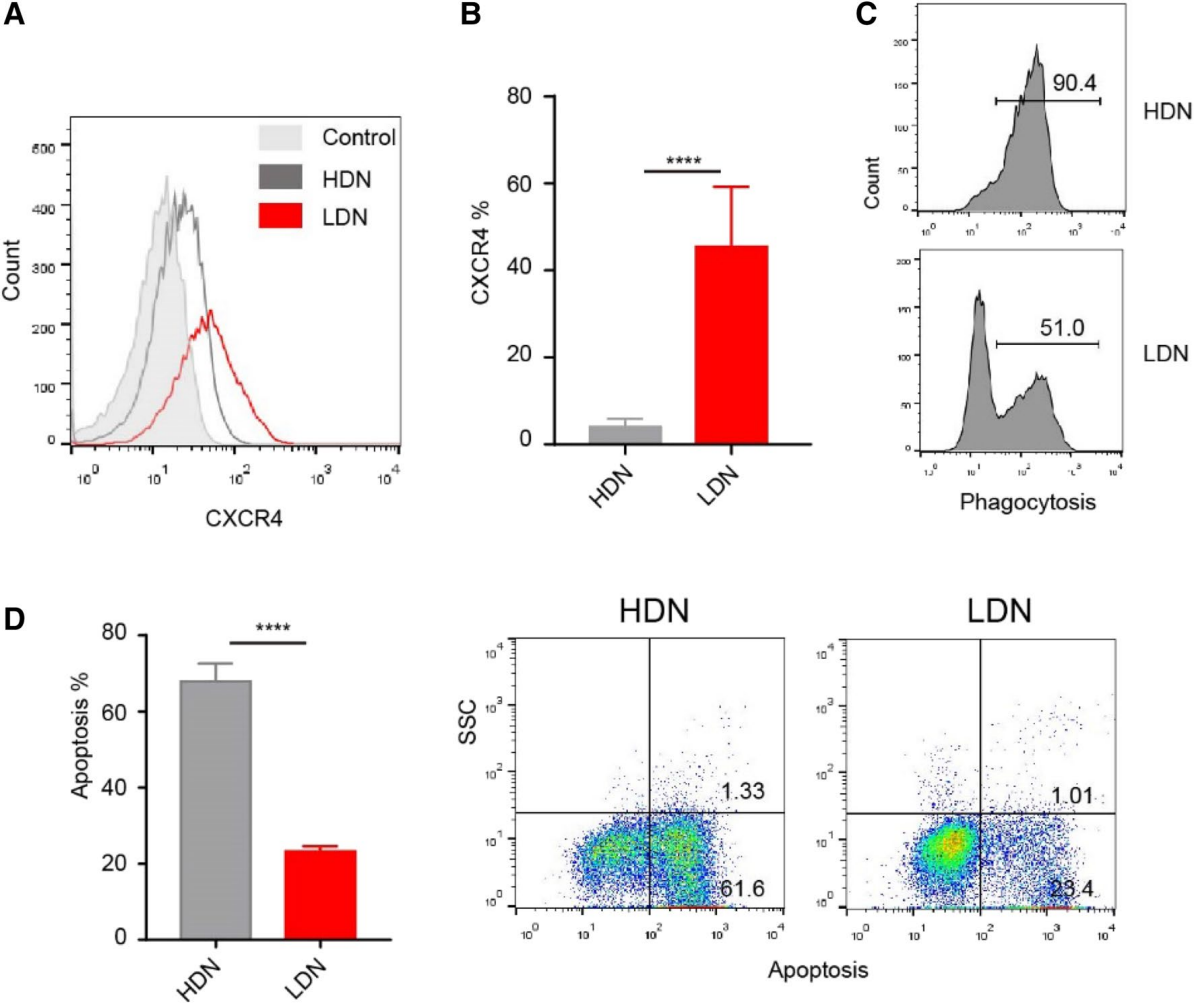
Changes in phagocytosis, apoptosis and surface molecular marker of LDNs in sepsis. (**A**, **B**) Expression of CXCR4 on HDNs and LDNs. (**C**) The phagocytosis of HDNs and LDNs analyzed using flow cytometry. (**D**) Apoptosis of HDNs and LDNs analyzed using flow cytometry.

Next, we studied the phenotype and function of LDNs. CXCR4 regulates the release of neutrophils from bone marrow and gradually disappears with the maturation of neutrophils^23^. The expression of CXCR4 increases with the age of circulating neutrophils. Neutrophils with high CXCR4 expression enter the bone marrow and are swiftly engulfed by local macrophages^24^. One study showed that CXCR4 expression is increased in freshly isolated blood neutrophils cultured in vitro for 4 h^25^. In the present study, we found that CXCR4 expression was higher in LDNs than in HDNs (Fig. 4A,B). We also measured phagocytosis (Fig. 4C) and apoptosis (Fig. 4D) in neutrophils and found that, compared with HDNs, the activity of LDNs was impaired, showing significantly decreased phagocytosis and delayed apoptosis.

### Decreased chemotaxis of LDN

**Figure 5:**
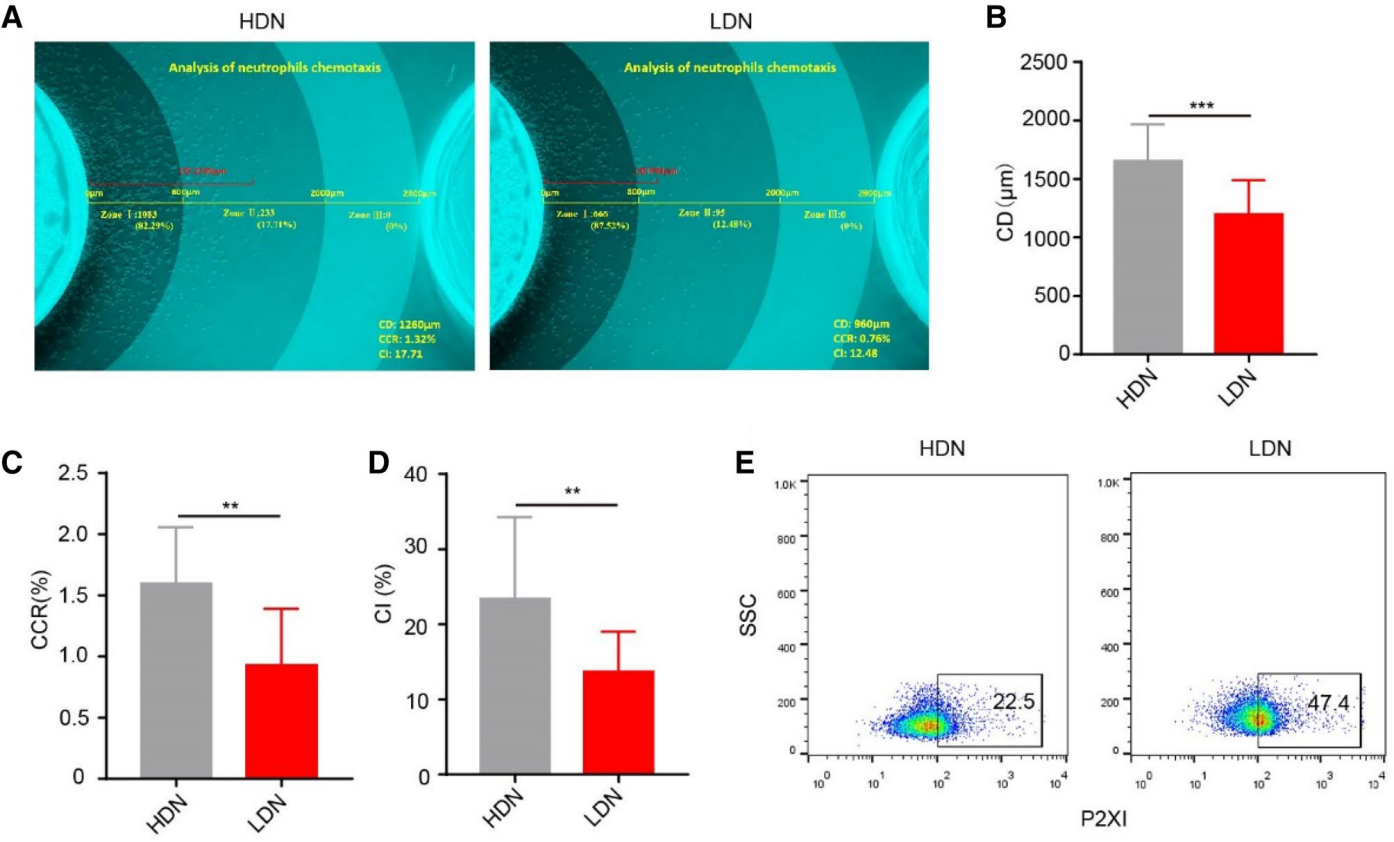
Reduced chemotactic function of LDNs. (**A**) Neutrophil chemotaxis toward fMLP was assayed. CD (chemotactic distance, μm), CCR (chemo cell ratio, cell number of zone I + II + III/Total cells in the side well, %), CI (chemo index, cell number of zone II + III/ Total number of chemotactic cells, %). (**B**–**D**) The evaluation indicators CD, CCR, and CI were significantly reduced in neutrophil chemotactic function evaluations of LDN. (**E**) The expression of P2X1 on HDNs and LDNs was analyzed using flow cytometry.

Neutrophils are recruited from the peripheral blood to the sites of infection through transmigration and chemotaxis. In sepsis, under the stimulation of various bacterial toxins and cytokines, the chemotactic direction of neutrophils is impaired, leading to decreased numbers of neutrophils arriving at the site of pathogen infection and increased cell infiltration in essential organs. These are among the fundamental reasons for immune dysfunction in patients with sepsis. Our previous study showed that neutrophils have a decreased chemotactic ability during sepsis^4^. In the present study, we used fMLP as the chemotactic agent and divided the chemotaxis area into three zones (zone I: chemotactic distance, CD < 800 μm; zone II: 800 < CD < 2000 μm; zone III: CD > 2000 μm) to perform a zonal analysis of chemotaxis images in detail. We used several parameters, CD, chemo cell ratio (CCR) and chemo index (CI) to evaluate the chemotactic function of neutrophils more comprehensively. It was difficult to isolate enough LDNs from the peripheral blood of healthy controls to complete chemotactic experiments. The chemotactic ability of neutrophils in sepsis patients was significantly reduced compared with normal density neutrophils in healthy volunteers. At the same time, we found that the chemotactic ability of LDNs was significantly weaker than HDNs in sepsis (Fig. 5A–D). The P2X1 receptor is an ATP-gated cationic channel receptor. Our previous studies showed that the P2X1 receptor is involved in the chemotactic inhibition of neutrophils during sepsis^21^. In the present study, we found that the expression of P2X1 in LDNs was significantly higher than in HDNs (Fig. 5E), possibly related to the decreased chemotaxis function of LDN.

## Discussion

Neutrophils have traditionally been considered homozygous terminally differentiated cells with essential functions. However, recent studies have shown that neutrophils have significant phenotypic heterogeneity and functional diversity, and they are an essential regulatory component of the immune response. Neutrophil dysfunction in sepsis is an essential factor that leads to the damage of the natural immune system and further aggravation of disease^26^. Monitoring sepsis using clinical criteria and peripheral white blood cell counts alone is inadequate because it does not allow for a detailed assessment of the immune system, especially the innate immune system.

At present, density gradient centrifugation is an essential method for the separation and purification of peripheral blood neutrophils. However, researchers often ignore LDNs in PBMCs. Due to the limitations of experimental methods, the operating costs of the instrument, and the staff’s operating skills, most methods used to study the function of neutrophils are rarely applied in clinical practice. Therefore, there remains a lack of an ideal detection system to reflect the changes in the functional activity of neutrophils in the process of sepsis.

Low-density granulocytes (LDGs) and LDNs are similar, and some studies even describe them as the same subset^11^. Most of the understanding of LDNs comes from various diseases such as asthma^27,28^, tuberculosis^29^, HIV infection^30^, psoriasis^19^, SLE^31^, and tumor^15,23^. However, there are very few studies on severe systemic infectious diseases. Morisaki et al.^22^ collected peripheral blood from patients with severe sepsis and generated three neutrophils with different densities using density gradients: HDN, intermediate-density neutrophils (IDN), and LDNs. In healthy patients, 76 ± 9% of neutrophils were HDNs, while LDNs accounted for only 4 ± 3% of all neutrophils. In contrast, only 8 ± 6% of HDNs and 40 ± 10% of neutrophils were LDNs in severely infected patients. In the present study, we found that the proportion of LDNs in PBMCs in sepsis patients reached 37.3 ± 5.11%, while the proportion in healthy volunteers was 3.96 ± 0.44%. CD10 expression was obtained during neutrophil maturation^13^. We further examined the expression of CD10 in sepsis neutrophils of different densities. We found that the majority of HDNs expressed CD10, and LDNs were composed of two subsets: CD66b^+^CD10^+^ and CD66b^+^CD10^-^. The proportion of the two subsets was significantly different among patients. This finding suggests that LDNs are composed of a mixture of mature and immature neutrophils in sepsis. Earlier studies showed that increased expression of CD10 in neutrophils was associated with improvement in patients with sepsis^22^, and this marker is therefore considered an indicator of the severity of clinical infection.

The source of LDNs remains unclear; however, four theories may shed light on the source. First, experimental factors (continuous centrifugation and washing) have been shown to activate HDNs, leading to degranulation and generation of LDNs^11^. Second, in the case of increased demand for circulating neutrophils, immature neutrophils can be absorbed into the circulation before the granules and nuclei mature, resulting in a relative increase in the proportion of LDNs^32^. Third, under the action of activators, LDNs would also be generated in the circulation due to the activation of HDNs, and the degranulation of circulating HDNs would lead to the relative increase of LDNs^29^. Fourth, in conditions such as cancer, there may be plasticity of HDNs to LDNs. Sagiv et al.^14^ found that mature HDNs transformed into LDNs under the action of tumor growth factor-beta in tumor-bearing mice. This conclusion has only been confirmed in animal tumor models and has not been verified in humans. Using our simple density gradient centrifugation, we did not find that LDNs could be induced. To understand which processes mediate changes in neutrophil density, we observed LDNs and HDNs in sepsis patients using transmission electron microscopy. Compared with HDNs, some LDNs are larger, have significantly reduced intracellular granules, and show substantial vacuolation with reduced granules. These results suggest that some LDNs in sepsis are derived from degranulated HDNs. A study showed an increase in LDNs in sepsis patients, and changes in LDNs can be used to determine whether neutrophils are activated to degranulation, thereby determining the severity of the infection^22^. LPS is a significant component of gram-negative bacteria's cell wall and is often selected as a stimulant for in vitro inflammatory studies^20^. LPS can induce degranulation of neutrophils in vitro, as reflected by increased plasma concentrations to MPO and MMP9^20^. Interestingly, the degree of degranulation after LPS stimulation positively correlated with the proportion of LDNs. These results suggest that some LDNs in sepsis may be due to neutrophil activation and degranulation.

The function of LDNs has been studied in a variety of disease models. Studies showed that HDNs provide anti-tumor protection, while LDNs have an immunosuppressive effect, causing tumors to grow out of control. These findings suggest that the ratio of HDNs to LDNs determines the contribution of neutrophils to the development or inhibition of tumors^14^. An oncological study demonstrated the immunosuppressive mechanism of LDNs. LDNs exhibited low apoptosis, chemotaxis, oxidative burst activity, and cytokine secretion, while these cells inhibited T cell proliferation and interferon-gamma production^23^. It was recently reported that the number of CD66b^+^ LDNs in the abdominal cavity significantly increased after gastric cancer surgery. These LDNs are prone to form NETs and selectively attach to cancer cells. These NETs can assist the aggregation and growth of abdominal free tumor cells^33^. However, in thrombocytopenia syndrome patients, the level of LDNs-induced proinflammatory cytokines was increased. Moreover, LDNs in severe fever with thrombocytopenia syndrome are toxic to endothelial cells, suggesting that LDNs may play an essential role in the induction of vascular injury^33^.

Previous studies showed that LDNs interchangeably known as low density granulocytes (LDGs). LDGs isolated from peripheral blood of patients with SLE showed significant proinflammatory responses. Gene sequence analysis showed 224 genes upregulated in LDGs in SLE patients, which were primarily related to serine proteases, bactericidal proteins, and molecules regulating inflammatory responses^12^. These cells may be involved in the pathogenesis of SLE by mediating proinflammatory responses that aggravate damage to terminal organs, enhance the ability of type I interferon synthesis, and kill endothelial cells. Although bacterial phagocytosis of LDGs decreased, the ability to form NETs was significantly enhanced. NETs have a potential role in externalizing autoantigens and activating the adaptive immune system^12,31,34^. LDGs in the peripheral blood of patients with psoriasis are also more likely to form NETS and externalize interleukin (IL)-17. IL-17 is a proinflammatory cytokine involved in the pathogenesis of several autoimmune diseases^19,35^. Therefore, some researchers believe that LDNs may be involved in the continuous chronic inflammation of the body, leading to autoimmune overplay^31,36^. In the present study, the immune function of LDNs in sepsis was not directly determined. Only one study showed that LDNs showed a decrease in β-glucuronidase activity and chemotactic ability during sepsis^22^. We showed that the immunological function of LDNs in sepsis was abnormal, characterized by decreased phagocytosis, delayed apoptosis, and reduced chemotaxis. These findings suggest that in sepsis, LDNs are less able to migrate to infected sites, have less bactericidal power, and may remain in the peripheral circulation for a longer time. This may mean a lack of ability to clear infectious agents and an increased susceptibility to infection. This is not the same as the function of aging neutrophils or autoimmune diseases in other studies, and the actual cause is not clear at present. However, one possible explanation is that different types of neutrophil could be generated at different times and in specific disease environments. Another possible reason is that sepsis LDNs are most likely neither purely aged nor purely immature PMNs, but rather represent a heterogeneous subset of neutrophils with special function. It is important to note that LDNs have not yet been labeled with a specific phenotype. Compared with normal neutrophils, LDNs in patients with acquired immunodeficiency syndrome expressed increased levels of CD15, CD33, CD66b, and decreased levels of CD62L, CD80, CD114, and CXCR4^17^. CXCR4 regulates neutrophil release from bone marrow and is associated with neutrophil maturation and senescence^23^. We found that the expression of CXCR4 in LDN was significantly higher than HDN during sepsis, suggesting that LDNs may be in different life stages.

In summary, we showed that LDNs are comprise a substantial proportion of neutrophils in sepsis, further enriching the concept of neutrophilic heterogeneity. We demonstrated for the first time that LPS increases the production of LDNs by promoting neutrophil activation degranulation. We also evaluated the characteristics and immune function of LDNs in sepsis in detail. By exploring the formation mechanism of LDNs and their abnormal immune function during sepsis, researchers can pay more attention to this neutrophil subset. Doing so may be helpful for the diagnosis and treatment of sepsis.
